# Labor market pathways to job quality mobility in the service sector: Evidence from the “Great Resignation”

**DOI:** 10.1016/j.rssm.2024.100962

**Published:** 2024-08

**Authors:** Tyler Woods, Dylan Nguyen, Daniel Schneider, Kristen Harknett

**Affiliations:** a Harvard Kennedy School, USA; b University of California, San Francisco, USA

**Keywords:** Job quality, Mobility, Labor market tightness, Service sector

## Abstract

Since the mid-1970s, there has been a sharp rise in the prevalence of “bad jobs” in the U.S. labor market, characterized by stagnant wages, unstable work schedules, and limited fringe benefits. Scholarly, policy, and public debate persists, however, about whether these jobs can serve as steppingstones to intra-generational job quality mobility or are instead “poverty traps.” While scholarship increasingly recognizes the multi-dimensional nature of job quality, prior research on intra-generational job mobility overwhelmingly estimates only wage mobility and generally focuses on estimating the degree of mobility, to the exclusion of the contexts and mechanisms that foster such mobility. We draw on new panel data collected from 8600 hourly service sector workers between 2017 and 2022 to estimate short-run mobility into good jobs, defined as paying at least $15/hour, having a stable work schedule, and offering paid sick leave, employer-sponsored health insurance, and retirement benefits. Overall, we find that mobility into such “good jobs” is low. However, we show that the rate of transition into “good jobs” is strongly conditioned by local labor market conditions: during the “Great Resignation” and in low state-month unemployment periods, nearly twice the share of workers transitioned to “good jobs” as in less favorable contexts, particularly workers who changed sector as opposed to staying at the same firm or taking new jobs in the service sector. Notably, during periods of labor market tightness, workers who stayed at the same employer had similar rates of mobility into “good jobs” as those who changed employers within the sector.

## 1 Introduction

Since the mid-1970s, there has been a sharp rise in the prevalence of “bad jobs” in the U.S. labor market, characterized by stagnant wages, limited fringe benefits, nonstandard work arrangements, and decreased job security (Autor and Dorn, 2013, Kalleberg et al., 2000, Kalleberg, 2009, Kalleberg, 2011, Lambert et al., 2012, Mischel et al., 2015, Pedulla, 2020). The increasingly precarious nature of work is particularly evident in the service sector (Kalleberg, 2009), which accounts for around 17 % of all jobs in the U.S. (Bureau of Labor Statistics, 2022). Indeed, service sector workers frequently experience low hourly wages, insufficient working hours, unstable and unpredictable schedules, and few critical benefits like paid sick leave (Bellew et al., 2022, Kuhlman and Schneider, 2023, Lambert et al., 2019, Sorensen et al., 2019), with significant negative consequences for workers’ well-being and economic security (Schneider and Harknett, 2019, Schneider and Harknett, 2021). In the face of such precarious working conditions, both public and academic discussions have often focused on the degree to which such jobs are “steppingstones” to better work or “dead ends” that trap workers in poverty (Andersson et al., 2005, Boushey, 2005, Campbell, 2012, Knabe and Plum, 2010, Mouw et al., 2024, Newman, 2020, Schultz, 2019).

While sociologists have recently brought a renewed focus to understanding broad trends in intra-generational occupational mobility (e.g., Jarvis & Song, 2017), the literature that takes up the more focused task of estimating mobility out of low-quality, precarious jobs has important limitations. First, this literature focuses almost exclusively on mobility out of low-*wage* jobs. While an important element of good jobs (Osterman & Shulman, 2011), wages are just one aspect of job quality. To date, though, scholarship has not systematically examined mobility into good jobs as defined multidimensionally, by wages as well as schedule stability, benefits, or more holistic measures of job quality, to the same extent it has examined mobility out of low-wage jobs. Some scholars, such as Jones and Schmitt (2016) and Gabe et al. (2018), have proposed multidimensional definitions of “good jobs”, with Gabe and colleagues showing that few workers in poor quality jobs transition into better jobs, but missing from these definitions is a measure that captures instability in workers’ schedules. This represents a notable oversight, given the association between unstable work schedules and a host of negative outcomes, including poorer sleep quality, increased psychological distress, and heightened work-family conflict (Luhr et al., 2022, Schneider and Harknett, 2019).

Second, much of the existing literature is focused on providing estimates of the degree of mobility but has devoted less attention to the contexts and mechanisms that foster such mobility, such as the macroeconomic labor market context and individual workers’ employment trajectories. Recent scholarship argues that labor market tightness plays an important role in improving job quality (Newman & Jacobs, 2023). This work builds on a limited body of evidence that suggests that job quality for low-wage workers can improve in tight labor markets (Andersson et al., 2005, Connolly et al., 2003, Newman and Jacobs, 2023). While much of this work is focused on wages, several studies examine the association between labor market conditions and other elements of job quality, such as work schedules (Finnigan, 2018, LaBriola and Schneider, 2020). However, a narrow focus on wages has so far characterized work that seeks to estimate the effects of the pronounced labor market tightness associated with the Great Resignation in the post-COVID period (Aeppli and Wilmers, 2022, Autor et al., 2023). For example, Autor and colleagues (2023) find that low-wage workers in the tight labor market following COVID-19 saw real wage growth, particularly young non-college workers. To date, no work assesses the effects of the Great Resignation-era labor market tightness on job quality more broadly defined.

Finally, some literature in sociology and labor economics asks how workers accomplish upward mobility from low wages, focusing on the employment pathways – such as by realizing wage gains through job retention (i.e., “job staying”) versus from job mobility (i.e., “job leaving”) (Andersson et al., 2005) – through which workers may transition out of “bad jobs.” This work finds that workers experience the greatest gains from switching jobs rather than staying at a job (Andersson et al., 2005, Autor et al., 2023). Much of the existing scholarship focuses on leaving low-wage sectors rather than leaving specific jobs, partly as a result of data limitations, which overlooks potential gains from moving to better jobs within the service sector (e.g., see discussion in Autor et al., 2023 of limitations of CPS data). Furthermore, little work examines how these relative gains from staying or leaving vary by labor market context (though, see Autor et al., 2023; French et al., 2005). Finally, like the literature on labor market context, this work has rarely examined the relative returns for job switching versus staying for elements of job quality beyond just wages.

In this paper, we address these methodological and theoretical gaps in the extant literature by examining improvements in job quality broadly defined in tight labor markets, including how this varies by individual workers’ employment trajectories. We offer three primary innovations to existing research. First, we move beyond the overwhelming focus of existing research on wages to include schedules and benefits, both individually and as a composite measure of “good jobs”, which we define as having 1) an hourly wage of $15/hour or greater; 2) low levels of schedule instability; and 3) retirement, health, and paid sick leave benefits. Second, we examine how labor market tightness shapes these dynamics, leveraging longitudinal, employer-employee matched data on service sector workers that spans before and during the COVID-19 pandemic and Great Resignation. The service sector is a strategic site to investigate this topic, as it has a large concentration of low-wage workers and was particularly affected during the COVID-19 pandemic and subsequent Great Resignation. Third, we examine how employment trajectories (i.e., staying or leaving a particular job or sector) shape mobility from bad jobs and how this varies by labor market context.

Using longitudinal data from The Shift Project from 2017 to 2022, we find that transitioning into a “good” job was relatively rare among the workers in our sample, but became much more common in tight labor markets, measured both as a dichotomous measure of the Great Resignation (compared to the prior period) and as a continuous measure of state-by-month unemployment rates. We find that workers also saw notable gains in hourly wages and schedule stability in tight labor markets, though we argue that focusing only on these individual metrics misses a more holistic picture of intra-generational job quality mobility. Next, we find that while workers generally saw the greatest returns to job quality from leaving their job for a job in a different sector, workers experienced significant boosts to job staying during tight labor markets, such that workers who stayed at their jobs during the Great Resignation were about as likely to transition to good jobs as those who switched to a new job within the service sector in the prior period.

## 2 Background

### 2.1 Mobility into good jobs

Work has become increasingly precarious since the 1970s (Kalleberg, 2009, Kalleberg et al., 2000). Though the origins of this precarity are complex and contested, many scholars point to the combined effects of both increasingly neoliberal policies that sapped worker power within firms and reduced the accessibility and generosity of the public safety net (Kalleberg, 2009, Rosenfeld, 2014) and of employers’ abandonment of the Fordist employment model (i.e., living wages, stable employment relationship) in favor of policies and practices that maximize shareholder value via short-term profits (Fligstein, 2001), particularly at the lower end of the wage distribution (Fligstein & Goldstein, 2022). The service sector is, in many ways, emblematic of the “bad jobs” arising from the increasingly precarious nature of work (Kalleberg, 2011), as it has the largest concentration of low-wage workers among all sectors (Osterman & Shulman, 2011), many of whom lack critical benefits like paid sick leave (Kuhlman & Schneider, 2023) and experience high levels of schedule instability and material hardship (Lambert, 2008, Schneider and Harknett, 2019, Schneider and Harknett, 2021).

Scholarship is divided on the implications of these “bad” jobs in the service sector for workers’ short- and long-term careers: they can either be “steppingstones” to better jobs or they can be “dead ends” that trap workers in poverty (Baranowska-Rataj et al., 2023, Bills et al., 2017, Boushey, 2005, Knabe and Plum, 2010, Mouw et al., 2024). The “dead-end hypothesis” suggests that low-wage jobs can become poverty traps if workers who begin in low-wage employment remain there, meaning that not only do these jobs have negative consequences for workers’ economic security, but they also have long-term implications for workers’ future careers (Baranowska-Rataj et al., 2023, Boushey, 2005, Knabe and Plum, 2010). This would arise if these “dead-end” jobs are disconnected from higher-paying jobs within the occupational structure due to the absence of skill-based mobility pathways (Escobari et al., 2021, Mouw et al., 2024). For example, using data from the Study of Income and Program Participation (SIPP), Boushey (2005) finds that prime-age workers (25−54) in minimum wage jobs are likely to get “stuck” there, particularly workers with stigmatized identities who lack structural power.

Meanwhile, the “steppingstone hypothesis” posits that workers can achieve upward mobility by using their low-wage employment as a starting point to find better work in a higher-paying sector, leveraging the work experiences and skills that they gained in these low-wage jobs to attain higher quality employment. This research challenges the idea that low-wage employment is always a bad place to start (Baranowska-Rataj et al., 2023, Knabe and Plum, 2010), highlighting the importance of both socioeconomic context and strong coworker networks to promoting upward mobility. More generally, a robust body of work finds that although mobility out of low-wage jobs has declined since the late 1990s, many workers do achieve upward mobility over time (Schultz, 2019). But for those who do exit low wages, such attainment is often temporary (Campbell, 2012).

Recent work by Mouw and colleagues (2024) offers one of the most rigorous adjudications between the “dead-end” and “steppingstone” hypotheses. Using data from the National Longitudinal Survey of Youth (NLSY) and the Study of Income and Program Participation (SIPP), the authors test whether working in low-wage jobs enables workers to move to higher-paid jobs in linked occupations, which they hypothesize is a function of acquiring transferable skills through work experience. They find strong support for the steppingstone hypothesis in certain low-wage jobs, offering evidence of a “positive effect of occupational work experience on the probability of upward wage mobility along mobility pathways to skill-linked destination occupations” (Mouw et al., 2024, p. 336). However, this work has several limitations that we build upon in our current study. First, the authors note in their conclusion that there is potential heterogeneity in their findings due to variation in labor market strength at the local and regional levels. Second, their study examines mobility out of low-wage jobs defined broadly, but there are likely important nuances within different sectors, such as the service sector, that pattern workers’ ability to achieve upward mobility. Third, they are exclusively focused on mobility out of low-wage jobs rather than conceptualizing job quality more broadly.

Indeed, the extant scholarship on upward mobility is overwhelmingly focused on workers’ attaining jobs with better wages, with little research exploring improvements in schedule stability or benefits, or holistic measures of good jobs. Yet job quality is multidimensional (Jones & Schmitt, 2016), and in conceptualizing what constitutes a good job, it is important to account for the myriad job features that matter for workers’ well-being and economic security. There is a limited but growing body of evidence on the consequences of temporal precarity, manifest in unstable and unpredictable work schedules, for workers’ health, well-being, work-life balance, and economic security (Lambert et al., 2012, Lambert et al., 2019, Luhr et al., 2022, Schneider and Harknett, 2019, Schneider and Harknett, 2021). But, despite the importance of this element of job quality for workers, little research has examined mobility into jobs with better schedules. Likewise, while scholars have pointed to the declining prevalence of health and retirement benefits as a key element of rising precarity (Kalleberg, 2011) and identified the importance of benefits like paid sick leave for workers (DeRigne et al., 2016), little work examines mobility into jobs with better benefits. The need for a broader and multi-dimensional definition of job quality is also underlined by policymakers efforts to complement long-standing minimum wage regulation with labor standards governing work schedule stability and paid leave (Williamson, 2023, Wolfe et al., 2018).

To date, though, very little research has attempted to put these various elements of job quality together into a holistic, composite measure of a “good” job. One key exception is the work of Janelle Jones and John Schmitt (Jones and Schmitt, 2016, Schmitt and Jones, 2012), who define a “good job” as one that pays at least $19/hour and has retirement and health benefits, elements they include both because of the substantive importance of these topics for job quality as well as because these items can be consistently measured over time. Drawing on longitudinal data from the Current Population Survey (CPS) from 1979 to 2010, the authors find that the overall share of U.S. workers in good jobs fell over the study period (Schmitt and Jones, 2012). Similarly, Gabe et al. (2018) develop a metric of job quality that encompasses hourly pay, desirability of the workweek, health insurance, and occupational prestige, and find that few workers transition to better jobs, and in fact are more likely to become unemployed than move up the job ladder.

While the complex and multidimensional nature of job quality precludes an easily agreed-upon composite measure, we argue that a strong definition of a “good job” must incorporate wages, schedules, and some core benefits. In this paper, we propose that a good job 1) pays at least $15/hour, in line with advocates’ push for $15 as a living wage for service sector workers (Greenhouse, 2022) and within the range of cutoffs that Mouw et al. (2024) use to define low-wage work (i.e., $14-$16); 2) has minimal schedule instability, which we operationalize using a schedule instability scale of five just-in-time scheduling practices (Lambert, 2008, Schneider and Harknett, 2019); and 3) has a critical suite of benefits: health insurance, retirement benefits, and paid sick leave.

### 2.2 How mobility is patterned by labor market tightness

In addition to rarely capturing a comprehensive measure of job quality, existing research on mobility out of “bad jobs” also rarely attends to the conditions under which mobility may be more or less likely. As Mouw et al. (2024) note, little work has “develop[ed] models that can identify the interaction between structural and individual-level factors using data on the career dynamics of workers over time” (p. 299). In particular, much of the existing work on mobility out of low wages is agnostic to a key structural factor: the labor market context in which individuals live and work. We know a great deal about the impact of slack labor markets (i.e., times of high unemployment) on individuals and families (e.g., Wilson, 2011), but significantly less scholarship has explored the effect of tight labor markets on low-wage workers. As Newman and Jacobs (2023) argue, the dynamics of tight labor markets are not simply the mechanical opposite of slack labor markets. In general, scholarship finds that tight labor markets can lead to wage increases. For example, Newman and Jacobs (2023) find that periods of tight labor markets, where the supply of jobs outpaces demand, can lead to increased wages and boost employment opportunities for low-wage workers. This boost is particularly impactful for Black and female workers (Bivens, 2021, Newman and Jacobs, 2023).

The labor market tightness following the COVID-19 pandemic was one such example of a tight labor market with the potential to improve job quality for workers. Recent research has documented how wage growth at the bottom of the distribution reduced income inequality (Aeppli & Wilmers, 2022), with especially large wage gains for young, non-college workers who changed employers and industries (Autor et al., 2023). In particular, using CPS data from 2015 to 2022, Autor and colleagues (2023) find that state-level labor market tightness predicts wage growth among low-wage workers and reallocation toward higher-paying sectors.

We know relatively little, however, about how tight labor markets shape job quality outside of their demonstrable effects on wages. A small body of work examines how labor market context shapes schedule stability. For example, LaBriola and Schneider (2020) study the extent to which work hour volatility is polarized by class and how this is moderated by worker power. The authors find that while low-wage workers experience disproportionately high volatility in work hours, reductions in marketplace bargaining power, operationalized as high state-level unemployment rates, reduce the class-based polarization of this volatility. However, this study does not examine whether labor market context is associated with individual-level improvements in schedule stability. Finnigan (2018) provides some insight into this question, documenting how the probability of experiencing work hour volatility increased during the Great Recession relative to the prior period. Furthermore, our understanding of how the effect of labor market context on upward mobility is patterned by workers’ employment pathways is likewise limited.

### 2.3 Pathways to mobility

Workers can achieve improvements in job quality primarily through two employment pathways: job mobility (i.e., “job leaving”) and job retention (i.e., “job staying”) (Andersson et al., 2005). While a large literature exists within labor economics on these pathways, little work focuses explicitly on the low-wage labor market (Holzer et al., 2004). Both pathways offer opportunities for improvement and setbacks for low-wage workers.

Turnover in low-wage jobs has mixed effects on earnings and does not always result in upward mobility (Holzer et al., 2004), as it can have a negative effect on earnings when that turnover is involuntary and leads to an unemployment spell (Andersson et al., 2005, Choper et al., 2022). But, turnover can also have a positive effect on earnings when it derives from workers voluntarily matching to better jobs (i.e., moving from low- to high-wage jobs) (Holzer et al., 2004, Neumark, 2002). For example, using an employer-employee matched longitudinal dataset of Illinois workers in the 1990s, Holzer et al. (2004) find that changing employers was an important source of earnings change, both for those entering and exiting low wages. Changing industries and/or occupations specifically is associated with a higher probability of exiting low-wage jobs (Boushey, 2005, Holzer et al., 2004, Schultz, 2019), although few studies capture individual workers’ job changes and how this affects wage changes, relying instead on information on sector changes with associated sector-level wages inferred to the worker level. Our understanding of how job leaving shapes improvements in other elements of job quality, like schedules or benefits access, is much more limited.

Workers can also realize gains from job retention due to the accumulation of additional skills (through on-the-job training) and additional experience (through increasing tenure and seniority) (Andersson et al., 2005, Holzer et al., 2004). For example, Holzer et al. (2004) finds that while job changers had the highest rate of partial or complete escape from low earnings (around 70 %), a sizable portion of job stayers (around 45 %) also experienced partial or complete escape. Andersson et al. (2005) note that the highest rate of escape from low earnings came from workers who changed jobs initially and then stayed with their employer. However, workers can also fail to advance within internal labor markets and remain stuck in low-wage jobs (Boushey, 2005).

Importantly, little work jointly examines how labor market tightness and employment pathways affect mobility from bad jobs. This is important because, as Andersson and colleagues (2005) note, “it is not job mobility alone that is likely to be successful, but rather job mobility in the presence of opportunities for work at higher-wage firms and jobs” (p. 79). Indeed, it’s possible that within a particularly tight labor market, workers could improve their job quality by either a) moving to a new employer that offers higher quality jobs (e.g., better wages, more stable schedules) to recruit workers and fill vacancies; or b) remaining at their job if their employer improves existing jobs to retain staff, reducing turnover by incentivizing workers to remain at the firm through higher wages or better schedules. One partial exception to this gap is Andersson et al. (2005), who find that strong labor markets can drive mobility from low wages. Distinguishing between gains from *job retention* (i.e., “job-stayers”) and gains from job mobility (i.e., “job-leaving”), they also find that job leavers have higher rates of transitioning out of low earnings relative to job stayers. However, the authors do not consider how the effects of different employment pathways on earnings vary across labor market contexts. The closest answer to this question comes from labor economics, where Autor et al. (2023) find that about half of the wage increase in the bottom of the wage distribution during the tight labor market post-COVID is associated with workers changing jobs rather than seeing wage growth in their same job. Similarly, Albagli et al. (2022) find that unemployment rates have a larger impact on new hires relative to job keepers. To our knowledge, no study has examined how labor market tightness and employment pathways together shape improvements in job quality beyond wages, such as to schedules or benefits.

## 3 Data and methods

### 3.1 Data

We draw on new longitudinal panel data collected via The Shift Project from a set of occupational cohorts of hourly workers employed at large firms in the service sector. These data allow us to define cohorts of workers who were employed in “bad jobs,” multi-dimensionally defined, at their baseline interview and then to track the mobility of these workers out of such jobs over time and to leverage geographic and temporal variation to examine how such mobility varied by labor market context and was enabled by different employment trajectories.

**Appendix Figure 1:**
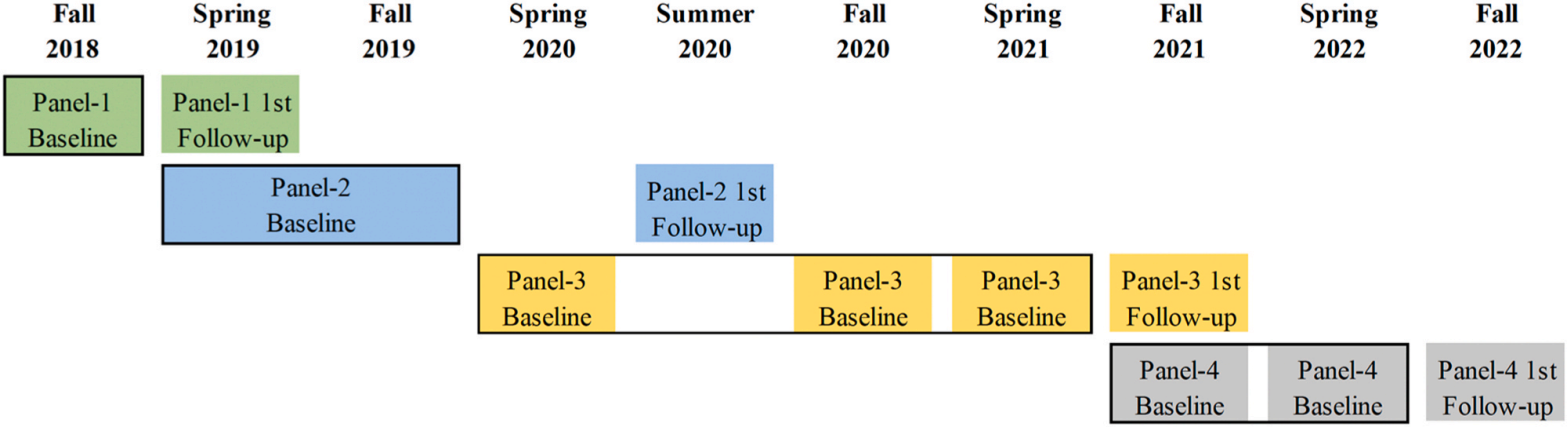
Overview of Shift Project Panel Data Design.

We assemble these occupational cohorts by recruiting hourly workers at large firms into twice-annual repeated cross-sectional surveys and then by empaneling respondents to those surveys by contacting them for up to three follow-up surveys over the 18 months following baseline. Using this approach, we have constructed four occupational cohorts who were interviewed at baseline between the Fall of 2017 and the Spring of 2022 and who were then followed over time over the period Spring of 2019 through Fall of 2022. Appendix Fig. 1 graphically depicts this staggered design.

We assemble the initial occupational cohorts using an online sampling and recruitment design. Specifically, the Shift Project recruits retail and food service-sector workers to complete web-based surveys using Facebook advertisements that target workers who are at least 18 years old, reside in the U.S., and are employed at large food service or retail employers. Facebook is one of the leading social media platforms, with a recent report estimating that nearly 70 % of U.S. adults are active on Facebook or Instagram (Auxier & Anderson, 2021). Potential respondents receive advertisements on their Facebook and/or Instagram feeds that offer a lottery incentive to complete a survey hosted through the Qualtrics platform. Workers who click through to the survey complete a consent and then are asked a set of detailed questions about their job quality and demographic attributes. These data have been used in a series of recently published papers (Choper et al., 2022, Schneider and Harknett, 2021).

Although concerns around selection bias may arise due to the Shift Project’s non-probability survey recruitment approach, research has found that statistically adjusting online, non-probability samples can produce similar distributions of measures and estimates of associations as probability-based samples (Gelman et al., 2016, Wang et al., 2015, Zhang et al., 2020). Furthermore, the Shift Project data are robust to a number of data validity checks, including benchmarking bivariate relationships in the Shift data against probability samples like the CPS and NLSY97 and directly assessing potential sources of unobservable bias (Schneider & Harknett, 2022). Detailed information on the data collection protocols can be found in Schneider and Harknett (2022).

Respondents to the cross-sectional surveys that were fielded between the Fall of 2017 and the Spring of 2022 were asked to provide email addresses and cell phone numbers and to consent to recontact. We then created four cohorts of these respondents who we attempted to recontact using email and text messages offering escalating survey incentives – starting with a $500 raffle and increasing to, at times, a $25 gift card – for completing a follow-up survey. These reinterviews were fielded between Spring 2019 and Fall 2022, between 3 and 25 months after baseline. At reinterview, respondents were asked to provide detailed information on their current employment status, job transitions since baseline, and, for those who were employed, current job quality. We draw upon one baseline and one follow-up observation per respondent.

Pooling across the four panels, 35 % of the 51,424 eligible respondents completed a reinterview. In Appendix Table A1, we model non-response to the reinterview as a function of demographic attributes and baseline job quality. We find no significant differences in nonresponse by gender or race, though we find that older workers are slightly more likely to complete a reinterview than younger workers. We also find significant but substantively small differences by baseline job quality: respondents with higher quality baseline jobs are more likely to complete a reinterview, but this effect size is small.

Our analysis sample then consists of 8600 respondents with a baseline interview between Fall of 2017 and Spring of 2022 and a reinterview between Spring of 2019 and Fall of 2022. We limit the sample to respondents who were reinterviewed between six and eighteen months after baseline to facilitate comparability across all panels. Additionally, we only include respondents who are employed at baseline and have complete data for all covariates. Analyses examining transitions to “good jobs” condition the sample on those who had “bad jobs” at baseline, defined below (N = 7738). Analyses that examine raw changes in job quality metrics loosen this restriction, but draw only on respondents with complete information on wages, schedules, and benefits at baseline and follow-up (N = 6685).

We provide descriptive statistics for the sample in Table 1. In general, this sample is majority female (74 %), majority white (78 %), and fairly young (43 % are under 29 years old). The average hourly wage for the sample is slightly under $15 an hour. Most respondents (79 %) remained at their employer at follow-up, while 15 % exited for an employer within the service sector and 7 % for an employer outside of the service sector, the most common of which were in the healthcare and education sectors.

**Table 1:** Sample Descriptive Statistics.

	Mean	SD
**Job Mobility Pathways**		
Same Employer	0.70	0.46
New Employer, Same Sector	0.18	0.38
New Employer, Different Sector	0.12	0.32
**Labor Market Tightness Measures**		
Observed During Great Resignation	0.49	0.50
State Unemployment Rate	6.38	3.03
**Job Quality Measures**		
Has a 'Good Job'	0.12	0.33
Unstable Scheduling (in past month)		
Pct. Cancelled Shift	0.10	0.31
Pct. Shift Timing Changed	0.57	0.50
Pct. Worked Clopeniong	0.32	0.47
Pct. Worked On-Call	0.18	0.38
Pct. Less than 2 Week Notice	0.51	0.50
Benefits Access		
No. of Benefits Received	3.22	2.70
Pct. Paid Sick Leave	0.57	0.49
Pct. Paid Vacation	0.68	0.47
Pct. Health Insurance	0.69	0.46
Pct. Dental Insurance	0.60	0.49
Pct. Paid Leave	0.35	0.48
Pct. Retirement Plan	0.52	0.50
Pct. Tuition Benefits	0.36	0.48
Pct. Childcare	0.08	0.27
Hourly Wage	15.00	5.80
**Demographics**		
Pct. Female	0.71	0.45
Race-Ethnicity		
White	0.80	0.40
Black	0.03	0.18
Hispanic	0.10	0.29
Other or 2 + Race	0.07	0.26
Age Group		
18 −19	0.09	0.28
20 −29	0.36	0.48
30 −39	0.16	0.36
40 −49	0.11	0.31
50 −59	0.15	0.36
60 −69	0.10	0.30
70 +	0.03	0.16
Pct. Students	0.22	0.42
Pct. Married/Living with Spouse or Partner	0.49	0.50
Pct. Has Kids	0.46	0.50
Pct. ESL Home	0.10	0.30
**Job Characteristics**		
Involuntarily Part-Time	0.18	0.38
*Job Tenure*		
Unemployed	0.09	0.29
Less than 1 Year	0.22	0.42
1 Year	0.14	0.35
2 Years	0.10	0.31
3 Years	0.09	0.28
4 Years	0.07	0.25
5 Years	0.05	0.21
6 + Years	0.25	0.43
*N*	8600	

### 3.2 Measures

#### 3.2.1 “Good jobs”

We employ the detailed data from the Shift Project to define a multidimensional measure of job quality. Specifically, we define a “bad job” as one that (1) pays less than $15 per hour, (2) has an unstable work schedule, and (3) lacks PSL leave, retirement benefits, and/or employer-provided health insurance. We assess (1) directly, by asking for workers’ hourly wage. We assess (2)–schedules–with a set of five items that measure schedule stability: whether respondents have experienced a) a canceled shift; b) a change in the timing of their shift; c) a clopening shift; d) an on-call shift; or e) less than two weeks of notice for their work schedule, all within the past month. We combine these five items into an index ranging from 0 to 5, then code respondents with a 0 or 1 on the schedule instability scale as experiencing “low schedule instability” and those with a 2 through 5 as experiencing “high schedule instability.” We assess (3)–benefits–using respondents’ reports of whether their employer provided them with paid sick leave, retirement, and with employer sponsored health insurance. Respondents who lack any of these elements of job quality are coded as being in “bad jobs” and those who exceed all thresholds as being in “good jobs.” In order to examine mobility out of “bad jobs,” we then follow prior research (Baranowska-Rataj et al., 2023, Holzer et al., 2004) in conditioning the analysis sample on being in a “bad job” at baseline.

We perform the same categorization exercise at follow-up survey for respondents who are employed. Employed respondents who are categorized as being in “good jobs” are coded then as having made the transition, while respondents who remain in “bad jobs” are coded as not having transitioned. Respondents who are unemployed at reinterview are also coded as not having transitioned to a “good job.”

Given that there is no consensus definition of a “good job,” in addition to our preferred measure of job quality, we also test the sensitivity of our estimates to a set of alternative definitions. First, we offer a number of alternative definitions of “good jobs” that tighten or loosen certain criteria, including 1) $15/hour, at least two weeks’ notice, and retirement, paid sick, and health insurance benefits; 2) $15/hour, at least two weeks’ notice and no changes in shift timing, and retirement, paid sick, and health insurance benefits; 3) $19/hour, 0 or 1 on schedule instability scale, and retirement, paid sick and health insurance benefits; 4) $19/hour, at least two weeks’ notice, and retirement, paid sick, and health insurance benefits; 5) $19/hour, at least two weeks’ notice and no changes in shift timing, and retirement, paid sick, and health insurance benefits; 6) $15/hour and retirement and health insurance benefits; and 7) $19/hour and retirement and health insurance benefits (i.e., the definition from Jones and Schmitt, 2016). We also model the raw changes in individual job quality measures between baseline and reinterview. For wages, we take the difference between respondents’ hourly wage across the two interviews. For schedule stability, we take the difference between respondents’ schedule instability scale (defined above) across the two interviews. For benefits, we take the difference between respondents’ benefits scale across the two interviews, which we define as an index, ranging from 0 to 8, of different benefits available from respondents’ employer: health insurance, dental insurance, paid sick leave, paid family leave, paid vacation, retirement plan, childcare, and tuition.

#### 3.2.2 Labor market context

We operationalize labor market context using two measures. First, we define a dichotomous measure by period in relation to the “Great Resignation.” While not defined precisely in the same way as recessions are, there is wide agreement that the labor market dynamics in the United States between April 2021 and July 2023 were distinct and characterized by very low unemployment rates and very high quit rates (Bunker, 2023, Gittleman, 2022). The Great Resignation then represents a period of pronounced labor market tightness. Respondents who were reinterviewed in this period are coded as “1” and those reinterviewed before or after are coded as “0.”

In addition to this dichotomous period-specific variable, we also operationalize labor market tightness using state-month unemployment rates calculated from the BLS Local Area Unemployment Statistics (LAUS), widely used data for characterizing local labor market unemployment (e.g., Autor et al., 2023). We merge state-month unemployment rates to the Shift Project micro-data using respondents’ reports of the state of their workplace and the date of their reinterview.

#### 3.2.3 Employment trajectories

Above and beyond the broad labor market context of unemployment, we also measure the specific employment trajectories by which individual respondents may transition into good jobs. We define a three-category variable as a function of respondents’ firm at baseline and their firm/sector at follow-up. First, we define one category as respondents who stay at the same firm between baseline and follow-up. Second, we define a group of respondents as those who transition firms but stay within the service sector. Third, we define a final category of respondents who change firms and transition out of the service sector. In models using this group of respondents, we omit those who transition to unemployment between baseline and reinterview.

#### 3.2.4 Controls

We control for a large number of worker characteristics measured at baseline. We control for respondents’ demographic characteristics: gender (male, female), race/ethnicity (white, non-Hispanic; Black, non-Hispanic; Hispanic; other or two or more races), school enrollment, marital status, parental status, and language other than English spoken at home. We also control for both job tenure at baseline and for involuntary part-time status at baseline, which we define as working fewer than 35 h per week at the focal job and reporting wanting more hours at that job. We additionally control for months since baseline survey, state and year fixed-effects, union density, and a state-year varying measure of prevailing minimum wage (from UC Berkeley’s Labor Center database).

### 3.3 Models

Our analysis proceeds in three steps. Our analysis file defines the key variables above by using both the baseline and follow-up data, but our file structure is a person-level data set, that is, we collapse the information from the two survey waves to define these change variables.

Using this file, we first estimate an OLS regression model predicting transition into a good job at follow-up, conditional on respondents being employed in a bad job at baseline. This model adjusts for the full set of controls described above. The key coefficient of interest in these models is months since baseline. Because the outcome is dichotomous, we can interpret the coefficients in this linear probability model as representing the probability change with respect to the dependent variable. We present predicted values of the share of workers transitioning to a “good job” across the observed values of months since baseline. In this, our estimates are similar to those of a synthetic cohort design, and we leverage variation in the precise timing of the reinterview to construct these estimates of transitions over time since baseline.

We next build on this core model to introduce our measures of labor market tightness. We first estimate the association between the Great Resignation and transitioning into a “good job.” We then estimate this association using our alternative operationalization of labor market tightness, state-month level unemployment rates. For both models, we generate predicted values of transition by Great Resignation exposure and by levels of state unemployment. Due to our data structure (i.e., only two observations per respondent) and our analytical focus (i.e., on the likelihood of transition rather than the duration to transition), we use OLS regression models rather than event history models.

Next, we examine the employment trajectories by which respondents transitioned into “good jobs” and how the impact of these pathways varied by labor market tightness. To do so, we interact our three-category measure of employment trajectory with each of our measures of labor market tightness. Finally, we also include versions of these models using continuous measures of change in wages, schedules, and benefits, rather than our dichotomous measures of “good jobs,” as the outcome.

## 4 Results

### 4.1 Mobility into good jobs

We begin by providing descriptive evidence on the degree of mobility that service sector workers experience out of jobs that are characterized by low pay, unstable and unpredictable work schedules, and a lack of core fringe benefits. A key contribution of our work is that the detail on job quality available in the Shift Project data allow for this multi-dimensional definition of a “good job.” However, there are reasonable alternative approaches to the precise definition of a “good job” and so we describe the sensitivity of our results to these alternatives below.

**Fig. 1:**
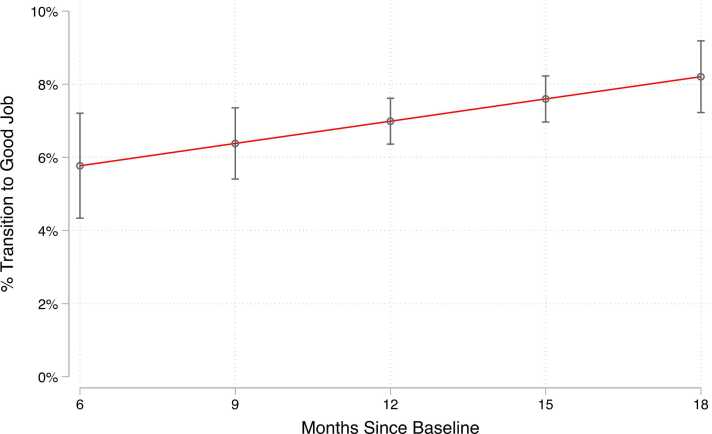
Predicted Probability of Transition to Good Job by Months Since Baseline Interview.

We first condition the baseline survey sample on workers who are not employed in “good jobs,” defined as hourly wage of at least $15/hour, a 0 or 1 on the schedule instability scale, and paid sick, retirement, and health benefits. Overall, we find that 90 % of the sample of hourly workers at baseline were not employed in good jobs so defined. In Fig. 1, we plot the share of these workers who had transitioned to a “good job” by month of follow-up. Here, we fit the transition probability using predicted probabilities from a regression model that adjusts for demographic and work characteristics, job quality measures, months since baseline, contextual measures, and state and year fixed effects. As described above, we pool respondents across panels in this analysis and leverage variation in the timing of reinterview to construct these predicted probabilities, similar to a synthetic cohort design. We find that transitioning into a good job is relatively rare among the hourly service sector workers in our sample. Six months following their baseline survey, just 6 % of workers had transitioned into a good job, with the share rising to just over 7 % by 12 months, and just over 8 % at 18 months after baseline.

**Appendix Figure 2:**
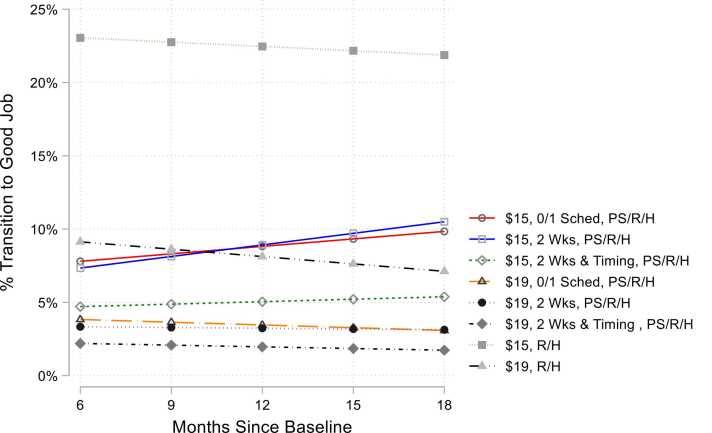
Predicted Probability of Transition to Good Job by Months Since Baseline Using Alternate Definitions of Good Job.

**Appendix Figure 3:**
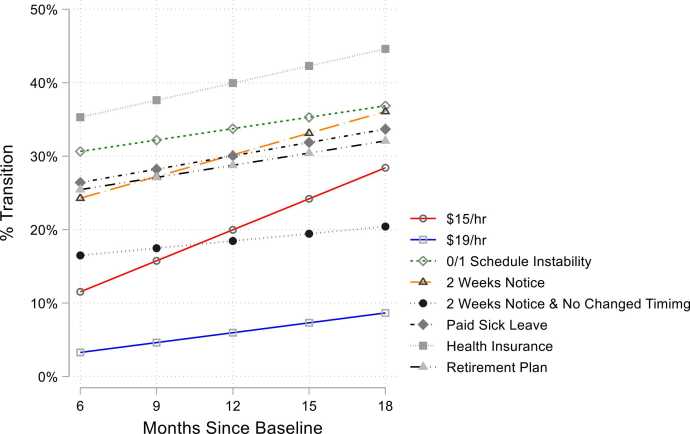
Predicted Probability of Transition to Good Job by Months Since Baseline Using Individual Job Quality Measures.

Appendix Fig. 2 shows the sensitivity of these estimates to alternative definitions of a “good job,” plotting the share of workers who had so transitioned at 6, 9, 12, 15, and 18 months by definition. Appendix Fig. 3 shows the sensitivity of these estimates to the individual job quality measures (wages, schedule stability, and benefits), rather than the holistic definitions. We find that the patterns of mobility are substantively the same across these alternate definitions of “good jobs,” as the coefficients remain significant and in the expected direction. The primary variation is in the degree of mobility, as the effect sizes are more conservative for more stringent definitions of job quality.

### 4.2 Labor market tightness and job mobility

The transition probabilities above pool across the experiences of workers from four panels, who were followed between 2017 and 2022 and across the country, between 6 and 18 months after their baseline survey. Yet, these workers experienced radically different labor market conditions depending on both the period and the place in which they were situated. We next estimate how the probability of transition into a “good job,” using our preferred definition above, varied by labor market tightness.

To do so, we estimate an OLS model of the transition to a “good job,” where the model controls for years of job tenure at baseline, months since baseline survey, respondents’ demographic characteristics at baseline (gender, race/ethnicity, school enrollment, marital status, parental status, and language other than English spoken at home) as well as for state fixed effects. We additionally control for time-varying measures of the state minimum wage and of union density.

**Fig. 2:**
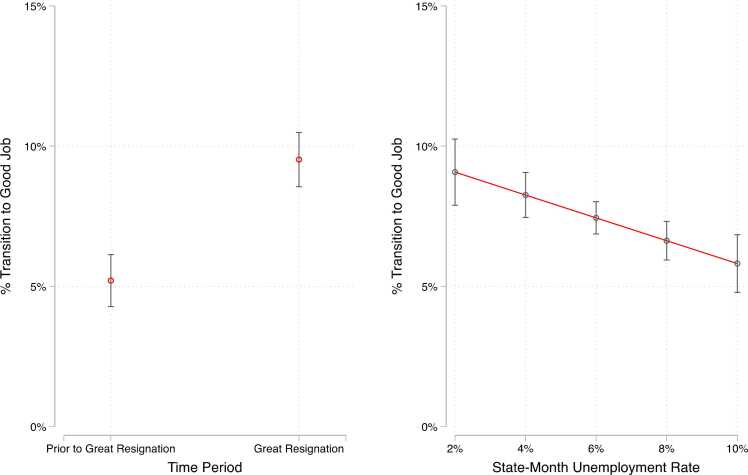
Predicted Probability of Transition to Good Job by Labor Market Context.

As shown in the top panel of Table 2, we estimate two models, each of which deploys a different measure operationalizing labor market tightness. M1 of Table 2 presents the regression results for our model that uses a dichotomous indicator for the period of the “Great Resignation” (respondents followed up between September 2021 through October 2022). We see that the probability of transition into a “good job” is nearly 4.5 percentage points higher, a near doubling of the chances (from 5 % on average to nearly 10 % on average), during the Great Resignation as compared to other periods between 2019 – 2022 (Fig. 2, left side). In M2 of Table 2, we model the transition to a “good job” as a function of state-month unemployment rates. As unemployment rises, the probability of transitioning to a good job declines (B = −0.004, p < 0.001). Where 9 % of respondents in low unemployment environments (2 % unemployment) transitioned to a good job by follow-up, that was true for just under 6 % of respondents in high unemployment environments (10 % unemployment) (Fig. 2, right side).

**Table 2:** Transition to Good Job Regressed on Labor Market Tightness.

	Including Unemployed at Follow-Up		Excluding Unemployed at Follow-Up	
	(1)	(2)	(3)	(4)
**Great Resignation** (ref: Prior to Great Resignation)	0.043^***^		0.046^***^	
	(0.008)		(0.009)	
**State-Month Unemployment**		−0.004^***^		−0.004^**^
		(0.001)		(0.001)
Observations	7738	7738	6713	6713

**Note**: Models includes controls for baseline demographic and job characteristics (gender, race/ethnicity, school enrollment, marital status, parental status, language other than English spoken at home, job tenure, involuntary part-time status), state fixed-effects, and time-varying measures of state minimum wage and union density.

Standard errors in parentheses

* p < 0.05,

* * p < 0.01.

* ** p < 0.001

In these models, we treat workers who transition to unemployment similarly to those who remain in bad jobs–both are considered to not have transitioned to a “good job.” In the bottom panel of Table 2, we reproduce our two models, but now exclude respondents who transition to unemployment from both the baseline denominator and from the risk set at follow-up. Our results remain robust to this specification. As above, the probability of transition to a good job is highest in tight labor markets, whether measured as the Great Resignation period (B = 0.046, p < 0.001) or as state-month unemployment rate (B = −0.004, p < 0.01).

**Appendix Figure 4:**
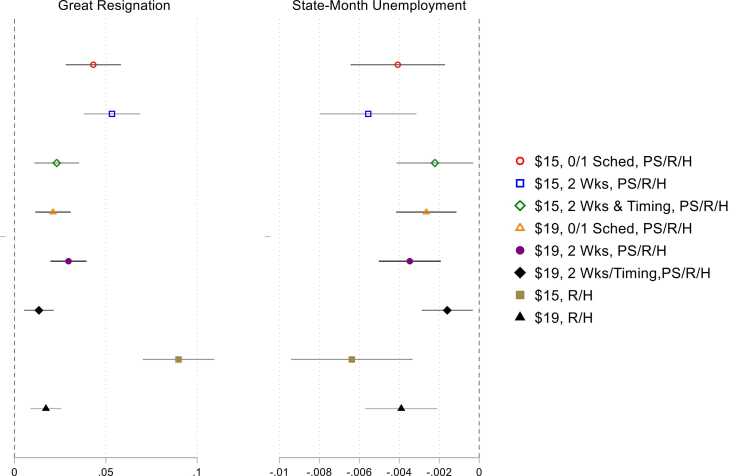
Coefficient Plot of Transition to Good Job Regressed on Labor Market Context using Alternative Definitions of Good Job.

In Appendix Fig. 4, we show the sensitivity of these estimates to the same set of alternative definitions of a “good job” as Appendix Fig. 2. We find that the coefficients for the Great Resignation and state-month unemployment rates for all definitions are in the same direction and in similar ranges of effect sizes, though the precise size of the effect varies slightly depending on the strictness of the definition.

### 4.3 Mechanisms of mobility

How did workers achieve the (albeit modest) upward job mobility that we document above? We examine how mobility into good jobs was produced differentially for workers who (1) stayed at the same employer between baseline and follow-up, (2) transitioned to a new employer within the service sector, and (3) transitioned to a new employer outside of the service sector. We then model how the job mobility returns to these trajectories differed by labor market tightness.

To do so, we re-estimate the models presented in Table 2, first introducing the three-category measure of employment trajectories (M1) and then interacting those measures with our two measures of labor market tightness (M2-M3). In these models, we exclude the 13 % of respondents who were employed in “bad jobs” at baseline and had transitioned to unemployment at follow-up because creating a unique fifth categories for these respondents would be entirely colinear with the outcome of non-transition to a good job.

**Fig. 3:**
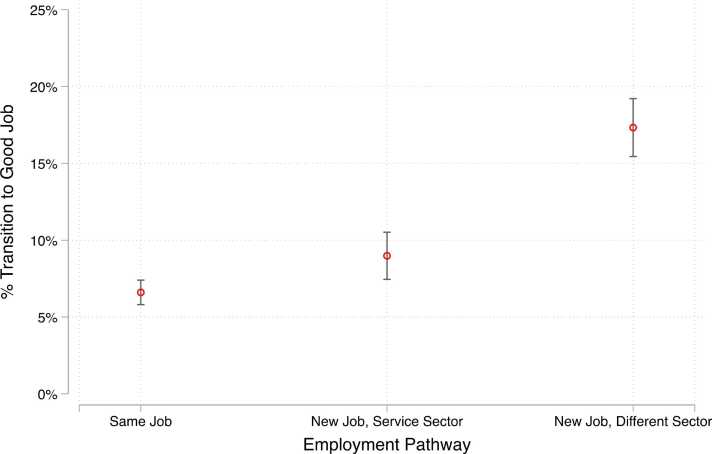
Predicted Probability of Transition to Good Job by Employment Pathway.

In M1 of Table 3, which includes the same set of controls as in Table 2, we find that, relative to staying at the same job, leaving a job is associated with a higher likelihood of transitioning to a good job, both for another job within the service sector (B = 0.024, p < 0.01) and for a job outside of the service sector completely (B = 0.107, p < 0.001). Specifically, while only 7 % of respondents who stayed in their same jobs transitioned to a good job, 9 % of respondents who moved to a new employer within the service sector and 17 % of respondents who moved to a new sector transitioned to good jobs. We plot these predicted probabilities in Fig. 3.

**Table 3:** Transition to Good Job Regressed on Labor Market Tightness by Employment Trajectory.

	(1)	(2)	(3)
**Employment Trajectory** (ref: Same Job)			
New Job, Service Sector	0.024^**^	0.034^**^	0.003
	(0.009)	(0.012)	(0.020)
New Job, Different Sector	0.107^***^	0.106^***^	0.124^***^
	(0.011)	(0.016)	(0.025)
**Great Resignation** (ref: Prior to Great Resignation)		0.044^***^	
		(0.010)	
**Employment Trajectory x Great Resignation Interaction**			
New Job, Service Sector x Great Resignation		−0.026	
		(0.017)	
New Job, Different Sector x Great Resignation		−0.005	
		(0.021)	
**State-Month Unemployment**			−0.004^**^
			(0.002)
**Employment Trajectory x State-Month Unemployment Interaction**			
New Job, Service Sector x State-Month Unemployment			0.003
			(0.003)
New Job, Different Sector x State-Month Unemployment			−0.003
			(0.003)
Observations	6713	6713	6713

**Note**: Models includes controls for baseline demographic and job characteristics (gender, race/ethnicity, school enrollment, marital status, parental status, language other than English spoken at home, job tenure, involuntary part-time status), state fixed-effects, and time-varying measures of state minimum wage and union density.

Standard errors in parentheses

* p < 0.05,

* * p < 0.01.

* ** p < 0.001

**Fig. 4:**
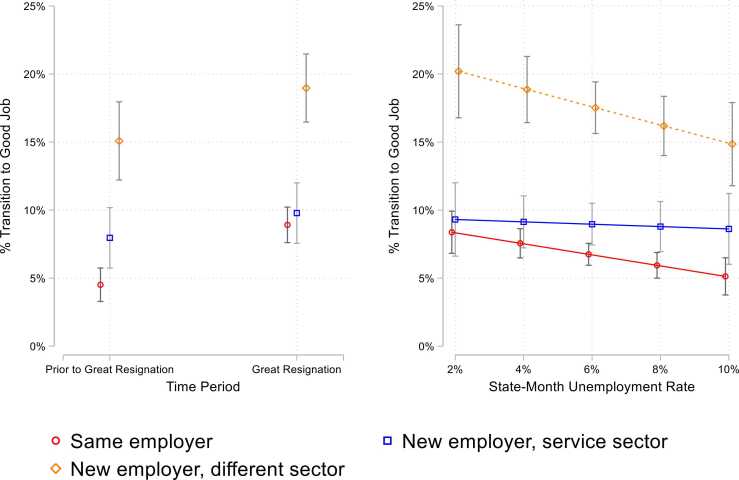
Predicted Probability of Transition to Good Job by Labor Market Context and Employment Pathway.

In M2-M3 of Table 3, we test the interaction of these trajectories with our two operationalizations of labor market tightness: the “Great Resignation” period (M2) and state-by-month unemployment rates (M3). As shown in Fig. 4 (left side), the Great Resignation offered a significant boost to respondents who stayed at their jobs: only 4.5 % of respondents who stayed at their jobs before the Great Resignation transitioned to a good job, compared to double (9 %) who transitioned during the Great Resignation. This suggests that tight labor markets offer a particular advantage to workers who remain in their jobs.

Workers who left their jobs for a new job within the service sector and for a new sector completely were likewise more likely to transition to a good job during the Great Resignation relative to the prior period. For example, 10 % of workers who moved jobs within the service sector transitioned to good jobs compared to 8 % who did so prior to the Great Resignation, while 19 % of workers who left the service sector during the Great Resignation did so for a good job, relative to 15 % prior to the Great Resignation.

Time-varying unemployment rates at the state level similarly suggest that tight labor markets are particularly beneficial to workers who remain in their same jobs (Fig. 4, right side). In M3 of Table 3, where we model state-by-month unemployment rates, we find that 8 % of workers who stay in their jobs in low state unemployment environments (2 % unemployment) transition to good jobs, compared to only 5 % in high state unemployment environments (10 % unemployment). This pattern holds for workers who left their jobs for new jobs in different sectors. For example, 20 % of workers in low state unemployment environments who left the service sector transitioned to a good job, compared to 15 % in high unemployment environments.

### 4.4 Robustness checks

We conduct several additional analyses to determine the robustness of our results to alternate specifications. First, in models that rely on state-month unemployment rates, we might worry that standard errors are correlated because individual workers are nested within specific geographic units (i.e., states). To overcome this limitation, we reran these models with standard errors clustered at the state level. Results are fully robust to this specification.

Second, we may also be concerned that there is some non-independence among workers who are employed at the same firm. We estimate all models with standard errors clustered at the employer level and, again, obtain similar results.

Third, because the cost of living can vary substantially across regions, and therefore the spending power of a given hourly wage (e.g., $15) also varies, we construct an alternate definition of a “good job” where the wage threshold is not universally $15, but rather is pegged to an hourly wage that is scaled to a state cost-of-living index developed by the Council for Community and Economic Research (C2ER). For example, in a state with a high cost of living (e.g., California), the necessary wage threshold for a “good job” is greater than $15, while in a state with a lower cost of living (e.g., Oklahoma), the wage threshold for a “good job” is lower than $15. Our results are fully robust to this specification.

**Appendix Figure 5:**
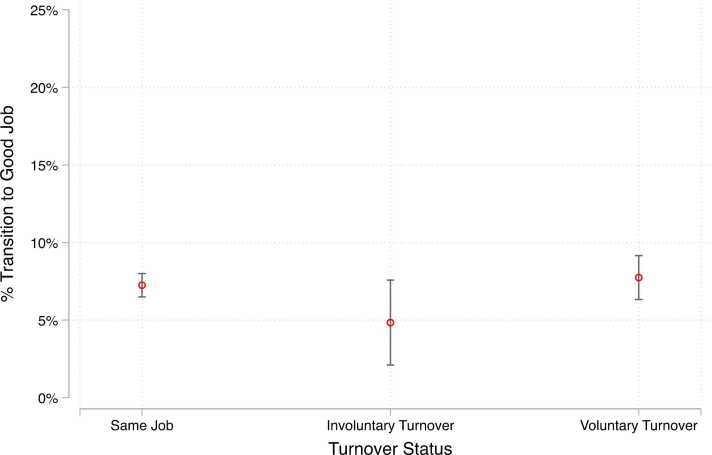
Predicted Probability of Transition to Good Job by Turnover Status.

Fourth, in addition to varying by labor market context and employment pathway, whether respondents transition to good jobs may vary based on whether respondents left their jobs voluntarily vs. involuntarily. For example, we may expect that workers who left their jobs voluntarily did so actively and strategically to maximize their wages, schedules, and benefits, whereas workers who left involuntarily (i.e., laid off or fired) may have experienced income or career disruptions. To test this idea, we re-estimate our primary models separately by whether respondents remained in their jobs, left voluntarily, or left involuntarily. We find a marginally significant relationship (p < 0.1) between transitions to good jobs and voluntary turnover status: respondents who left their jobs involuntarily are less likely to transition to a good job at reinterview (around 5 %) than those who left their jobs voluntarily (around 5 %) and those who remained in their jobs (around 7 %) (Appendix Fig. 5). This suggests that there is a penalty primarily for being fired or laid off, but not necessarily a large boost for those who quit relative to those who stay.

We then estimate our models with an interaction effect between labor market tightness and voluntary turnover status, which we find is positive and statistically significant (B = 0.056, p < 0.01). The effect size of tight labor markets is larger for those who voluntarily left their jobs relative to those who stayed in their jobs and those who left involuntarily. For example, taking predicted probabilities, 11 % of respondents who voluntarily left their jobs were able to transition to good jobs during the Great Resignation, compared to 9 % of those who stayed in their jobs and 8 % of those who left involuntarily. These analyses suggest that workers who can leverage the heightened worker power in the labor market to leave their jobs voluntarily are the most likely to transition to good jobs.

### 4.5 Changes in job quality measures by labor market context

Next, we model the raw changes in hourly wages, schedule instability, and benefits across our two measures of labor market tightness. In doing so, we highlight the additive value of using a composite “good jobs” measure rather than individual metrics of job quality like hourly wages. Overall, we find that workers experienced substantial gains to hourly wages and schedule stability during periods of tight labor markets, but received less of a boost in the number of benefits that their jobs offered. However, this could be due to the short follow-up period, as some workers may not have been eligible for benefits by the reinterview survey.

In M1-M2 of Table 4, we quantify the raw changes in hourly wages, schedule instability, and benefits across our two measures of labor market tightness.

**Table 4:** Changes in Job Quality Measures Regressed on Labor Market Tightness.

	Change in Wages		Change in Schedules		Change in Benefits	
	(1)	(2)	(3)	(4)	(5)	(6)
**Great Resignation** (ref: Prior to Great Resignation)	1.253^***^		−0.078		−0.124	
	(0.107)		(0.044)		(0.074)	
**State-Month Unemployment**		−0.117^***^		0.026^***^		−0.018
		(0.017)		(0.007)		(0.012)
Observations	6685	6685	6685	6685	6685	6685

**Note:** Models includes controls for baseline demographic and job characteristics (gender, race/ethnicity, school enrollment, marital status, parental status, language other than English spoken at home, job tenure, involuntary part-time status), state fixed-effects, and time-varying measures of state minimum wage and union density.

Standard errors in parentheses

* p < 0.05,

* * p < 0.01.

* ** p < 0.001

**Fig. 5:**
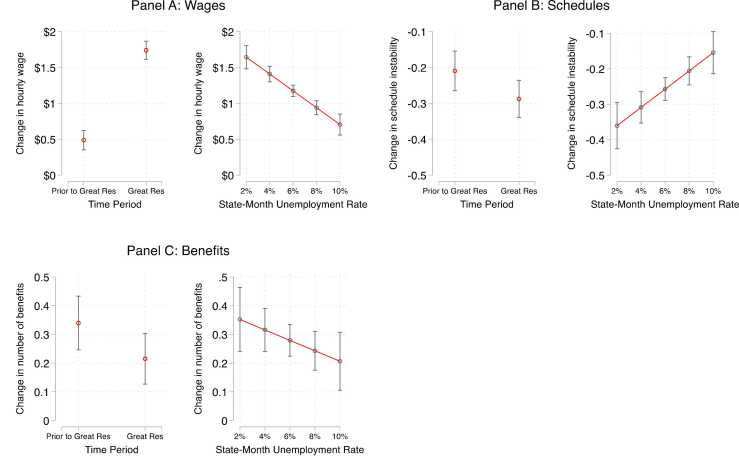
Predicted Probabilities of Change in Job Quality Metrics by Labor Market Context.

Beginning with wages, we find a significant and positive relationship between the Great Resignation (Fig. 5, Panel A) and changes in hourly wages (B = 1.253, p < 0.001). Taking predicted probabilities, before the Great Resignation, respondents saw an average gain of around $0.49, while workers during the Great Resignation saw an average gain of almost $1.74. This pattern is similar for state unemployment rates (B = −0.117, p < 0.001). This finding mirrors prior work on wage gains for low-wage workers during moments of labor market tightness.

Workers also saw some reductions in schedule instability in tight labor markets, though the size and significance of the effect varies across our measures. As shown in Fig. 5, Panel B, we find that workers experienced modest but marginally significant gains to schedule stability during the Great Resignation relative to the prior period (B = −0.078, p < 0.1), but substantial and significant gains to schedule stability during periods of low state unemployment (B = 0.026, p < 0.001). Examining marginal effects, we find that workers in low state unemployment periods saw reductions of around 0.36 on the schedule instability scale, while those in slacker state labor markets saw reductions of 0.15.

Finally, and somewhat surprisingly, we find that workers in our sample experienced a smaller boost in the number of benefits their jobs offered during the Great Resignation, although this coefficient is only marginally significant (B = −0.12, p < 0.1). For example, we find that workers before the Great Resignation saw a gain of 0.34 benefits, while respondents during the Great Resignation saw a gain of only 0.22 benefits (Fig. 5, Panel C). We do not find any significant differences based on state unemployment rates.

### 4.6 Changes in job quality measures by labor market context and employment pathway

In M1-M2 of Table 5, we evaluate how these gains in job quality during tight labor markets varied by employment trajectories. We begin by testing how changes in wages, schedules, and benefits vary by employment trajectory, and then interact these trajectories with our labor market tightness measures.

**Table 5:** Changes in Job Quality Regressed on Labor Market Tightness by Employment Trajectory.

	Change in Wages			Change in Schedules			Change in Benefits		
	(1)	(2)	(3)	(4)	(5)	(6)	(7)	(8)	(9)
**Employment Trajectory** (ref: Same Job)									
New Job, Service Sector	0.311^**^	1.024^***^	−0.968^***^	−0.522^***^	−0.484 ^***^	−0.446 ^***^	−0.108	−0.093	−0.337
	(0.113)	(0.159)	(0.254)	(0.045)	(0.065)	(0.103)	(0.079)	(0.113)	(0.180)
New Job, Different Sector	2.414^***^	2.764^***^	1.956^***^	−1.024^***^	−0.877 ^***^	−1.287 ^***^	0.164	0.152	0.199
	(0.135)	(0.199)	(0.312)	(0.055)	(0.081)	(0.126)	(0.095)	(0.142)	(0.221)
**Great Resignation** (ref: Prior to Great Resignation)		1.497^***^			0.021			−0.130	
		(0.117)			(0.048)			(0.083)	
**Employment Trajectory x Great Resignation Interaction**									
New Job, Service Sector x Great Resignation		−1.447 ^***^			−0.073			−0.017	
		(0.215)			(0.088)			(0.153)	
New Job, Different Sector x Great Resignation		−0.811^**^			−0.259*			0.038	
		(0.262)			(0.107)			(0.187)	
**State-Month Unemployment**			−0.150 ^***^			0.021^**^			−0.023
			(0.018)			(0.007)			(0.013)
**Employment Trajectory x State-Month Unemployment Interaction**									
New Job, Service Sector x State-Month Unemployment			0.205^***^			−0.012			0.037
			(0.037)			(0.015)			(0.026)
New Job, Different Sector x State-Month Unemployment			0.070			0.042*			−0.006
			(0.045)			(0.018)			(0.032)
Observations	6685	6685	6685	6685	6685	6685	6685	6685	6685

**Note**: Models includes controls for baseline demographic and job characteristics (gender, race/ethnicity, school enrollment, marital status, parental status, language other than English spoken at home, job tenure, involuntary part-time status), state fixed-effects, and time-varying measures of state minimum wage and union density.

Standard errors in parentheses

* p < 0.05,

* * p < 0.01.

* ** p < 0.001

**Fig. 6:**
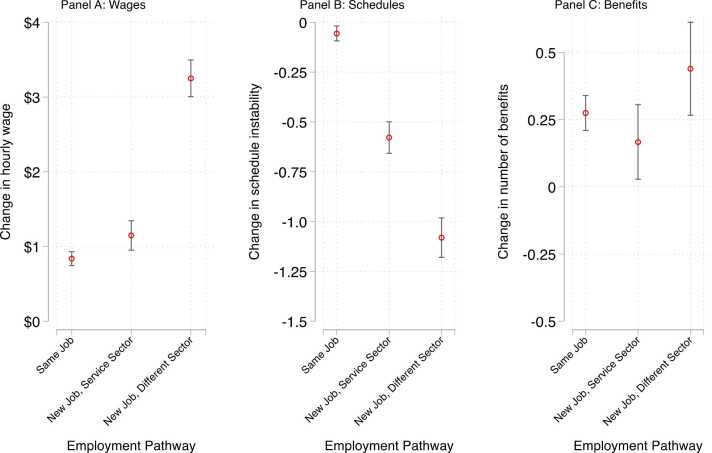
Predicted Probabilities of Change in Job Quality Metrics by Employment Pathway.

Beginning with the association between employment pathways and wage gains (Fig. 6, Panel A), we find that workers who left the service sector experienced the highest changes in hourly wages (B = 2.414, p < 0.001), followed by those who left their jobs for another job in the service sector (B = 0.311, p < 0.01).

**Fig. 7:**
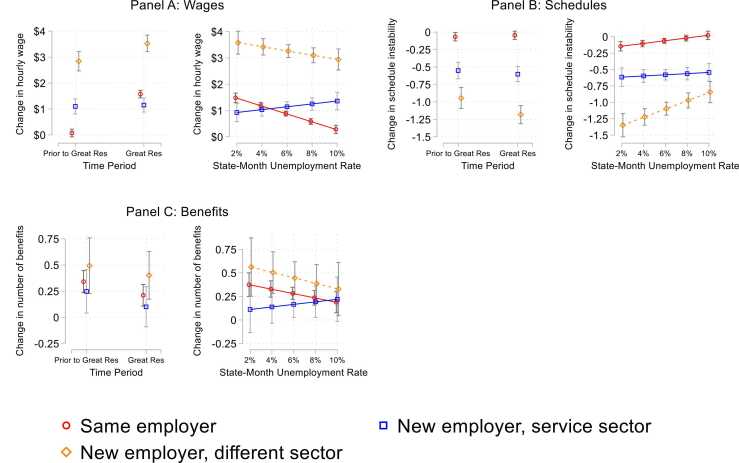
Predicted Probabilities of Change in Job Quality Metrics by Labor Market Context and Employment Pathway.

As shown in Fig. 7, Panel A, workers who stayed in their jobs experienced the greatest boost in wages during tight labor markets: prior to the Great Resignation, job stayers saw an average wage change of $0.076, whereas during the Great Resignation job stayers saw wage gains of around $1.57. These gaps between the labor market periods narrowed for those who left their jobs but still remained. For example, workers who changed sectors before the Great Resignation saw an average gain of around $2.84, while those who changed sectors during the Great Resignation saw wages gains of $3.53. Job leavers saw substantial gains in schedule stability relative to job stayers (Fig. 6, Panel B), including both those who left for other jobs in the service sector (B = −0.522, p < 0.001) and those who changed sectors (B = −1.024, p < 0.001).

Workers who left their jobs for a new sector saw an added wage boost during the Great Resignation (decline of 1.18 on the schedule instability scale) relative to the prior period (decline of 0.94), as well as an added boost during periods of low state unemployment (decline of 1.35) relative to high state unemployment (decline of 0.84) (Fig. 7, Panel B).

As shown in Fig. 6, Panel C, we find that workers who leave their jobs for a new employer in the service sector saw a small but marginally significant increase in the number of benefits they receive from their employer (0.44 increase in number of benefits). We find no significant differences in the effect of job leaving on changes in benefits by labor market tightness. It is possible that this difference in significance is partly driven by workers’ preferences regarding the aspects of job quality that they value. For example, descriptive analyses from Shift Project data show that more workers believe that level of pay and schedule predictability are somewhat or extremely important parts of a good job (96 % and 94 %, respectively) relative to benefits (81 %). Thus, workers may be more willing to leave their jobs for ones that offer improvements in wages and schedules.

Taken together, this analysis of changes in individual job quality metrics across labor market contexts and employment pathways shows that service sector workers experienced gains in wages and schedule stability in tight labor markets, particularly thoughts who left their jobs. However, we argue that focusing only on these individual metrics misses a broader, more holistic picture of job quality mobility. For example, while we document that workers saw these gains in wages and schedules, our analyses of “good jobs” highlights that these gains occur in tandem with rather than in isolation from one another.

## 5 Discussion and conclusion

The low-wage retail and food service sector comprises nearly 1 in 5 jobs in the contemporary U.S. economy. Across multiple dimensions, the vast majority of these jobs are quintessential “bad jobs,” characterized by low wages, few benefits, and unpredictable and unstable work schedules. In this paper, we take up the question of whether these jobs are dead ends that trap workers in poverty with little chance for upward mobility, or how often and under what conditions these jobs serve as steppingstones to better occupational opportunities.

We draw on newly available longitudinal data from 8600 service sector workers employed in the typical “bad jobs” of this sector and follow these workers for up to 18 months to assess their short-term occupational mobility. Our data allows us to extend prior literature in three noteworthy ways. First, we extend prior research, which has largely focused on wage mobility, by encompassing multiple dimensions in our definition of good jobs – wages, fringe benefits, and precarious or stable work scheduling conditions. By also showing results for these individual job quality measures, we highlight the additive value of using a multidimensional measure. Second, we build on work by Mouw et al. (2024) by considering the contexts and mechanisms that foster upward mobility. Specifically, the period covered by our data allows us to compare job mobility during adjacent time periods with dramatically different labor market conditions. Third, we distinguish among employment pathways to good jobs: through improved conditions over time at one’s original job, through job changes within the service sector, or through departures from service sector jobs for job opportunities in a new sector. To our knowledge, no study has examined how labor market tightness and employment pathways together shape improvements in job quality beyond wages, like schedules or benefits.

In general, we find that workers who began in bad jobs in the service sector experienced more upward mobility to good jobs when they changed jobs, especially in favor of opportunities in a new sector, compared with those who remained in the same position over time. This result aligns with earlier research by Andersson et al. (2005), which find that those who transition to new job opportunities are more likely to experience upward wage mobility compared with those who remain in their jobs. We extend this result with more recent data and by expanding the focus from wages as the sole indicator of a good job to include fringe benefits and stable and predictable work schedules.

Overall, workers experienced more upward mobility during the strong economy of the Great Resignation and in states with tighter labor markets based on their low unemployment rates. These results are consistent with findings from the Current Population Survey, which found that state-level labor market tightness is associated with wage growth among low-wage workers and mobility to higher-paying industry sectors (Autor et al., 2023). Our results also are consistent with and extend findings from Finnigan (2018), which found that work hour volatility increased during the slack labor market of the Great Recession relative to the prior, stronger labor market period.

Our analysis uniquely combines an examination of intra- versus inter-job mobility to good job conditions under different labor market conditions. A striking finding revealed from this approach is that the strong economy benefitted those who remained in their jobs even more so than those who changed jobs. Strong economies increase worker power, and during the Great Resignation, this translated to appreciable improvements in working conditions, particularly better wages and better schedules for workers who remained with their original employer. For those who remained in their jobs, the rate of upward mobility to good jobs during the Great Resignation period was double that of the prior period in which the labor market was less strong. It is also possible that employers improved job quality for their employees as a retention strategy or in response to workers’ attempts to bargain using outside offers.

The large gains for job-stayers contrast with findings reported by Autor et al. (2023), which found that about half of the wage increase in the bottom of the wage distribution during the tight labor market post-COVID is associated with workers changing jobs rather than seeing wage growth in their same job. Similarly, Albagli et al. (2022) found that unemployment rates have a larger impact on new hires relative to job keepers. Our differing results likely stem from our focus on those originating in bad jobs and our broader definition of good jobs compared with these prior studies.

Putting these results in a broader context, however, transitioning from a bad to a good job was the exception, not the rule, for all employment paths (staying or leaving a job and staying in or leaving the service sector), and during or prior to the Great Resignation period. Overall, 92 % of those who began in a bad job remained in a bad job 18 months later. Even in the best-case scenario, during the strongest labor market conditions, mobility to a good job was limited to 8 % of those who began in bad jobs. Furthermore, although we label these jobs as “good” jobs relative to other low-wage employment, it bears noting that these jobs are still at the lower end of the job quality distribution, and are perhaps more accurately titled “better” jobs.

In interpreting these novel findings, some sample features and data limitations should be kept in mind. First, by design our sample primarily comprised those who began in bad jobs in the service sector, with the aim of gauging the extent and conditions under which these workers could make the leap to good jobs. Therefore, by design, our analysis does not capture downward mobility from good jobs or upward mobility from good jobs to better jobs. For example, future work could seek to document the prevalence and predictors of downward mobility, including if a worker who attained a “good job” during a tight labor market reverts to a “bad” job in a slack labor market. Second, our follow-up period is relatively short, tracking upward mobility to good jobs over an 18-month period. The limited follow-up period means that we are not observing rates of upward mobility to good jobs over the longer-term, and we are also unable to capture whether good-jobs gains are sustained over the longer term. Third, due to sample size restrictions, we only draw on one follow-up survey per respondent, which precludes an analysis of multi-step transitions.

Prior research suggests that mobility from low-wage jobs tends to be fleeting (Campbell, 2012), and we cannot speak to whether those who transitioned to good jobs will be able to maintain and build upon this upward mobility over the longer term, particularly when labor market conditions weaken. Third, we reported on patterns of non-response that could introduce some bias into our estimates. In particular, those who responded to follow-up surveys tended to be younger and to have somewhat better job quality compared with non-respondents. Therefore, we may be slightly overestimating the extent of upward job mobility, if those with relatively better bad jobs have an easier time accessing good jobs than their counterparts with the worst bad jobs who were more likely to attrite from the panel.

These results have notable implications for policy and practice. First, our multidimensional approach to good jobs emphasizes the need for both policymakers and employers to move beyond the singular focus on wages, instead considering the importance of factors such as schedule stability and fringe benefits to workers. For example, the passage of secure scheduling laws in cities like Seattle is a strong step in this direction. Second, our findings on job quality improvements among job stayers in tight labor markets suggest that offering higher wages, more stable schedules, and more generous benefits may be an effective retention strategy for firms in periods of low unemployment, encouraging workers to realize gains in their existing job rather than seeking better options in the labor market. Third, our respondents consistently achieved the greatest improvements in job quality by leaving the service sector entirely. However, workers might be more likely to remain in the sector if there were stronger internal career ladders. Firms in the service sector should consider creating more opportunities for upward career advancement for hourly workers, such as pathways to management or the corporate workforce.

In sum, with newly available panel data that spans the period before and during the Great Resignation and includes measures of multiple dimensions of job quality, our paper has made several novel contributions to the literature. We show that changing jobs and leaving the service sector are generally more common paths to upward mobility compared with longer tenure at one’s original service sector employer. We also show that strong labor markets increase the rate of transitioning to good jobs for each of three employment paths: staying with one’s original employer, moving to a new service sector job, or moving to a job outside of the service sector. Further, strong economies relative to weaker economies pay the largest dividends for workers who remain in their original positions. The results demonstrate that service sector jobs do not inevitably become dead-end poverty traps, and switching jobs and strong economies make a noticeable difference in increasing upward mobility. However, even under the best labor market conditions, most workers who begin in bad jobs remain in bad jobs 18 months later.

## CRediT authorship contribution statement

**Dylan Nguyen:** Writing – review & editing, Writing – original draft, Visualization, Investigation, Formal analysis, Data curation, Conceptualization. **Tyler Woods:** Writing – review & editing, Writing – original draft, Visualization, Validation, Supervision, Methodology, Investigation, Formal analysis, Data curation, Conceptualization. **Kristen Harknett:** Writing – review & editing, Writing – original draft, Supervision, Project administration, Methodology, Investigation, Funding acquisition, Conceptualization. **Daniel Schneider:** Writing – review & editing, Writing – original draft, Supervision, Resources, Project administration, Methodology, Investigation, Funding acquisition, Conceptualization.

## Declaration of Competing Interest

The authors report no conflicts of interests.

## Figures and Tables

**Table A1:** Modeling Non-Response to Reinterview as a Function of Demographic Characteristics and Baseline Job Quality.

	(1)
	Nonresponse
**Demographic Characteristics**	
Gender (ref. Male)	0.003
	(0.006)
Race-Ethnicity (ref. White Non-Hispanic)	
Black Non-Hispanic	−0.000
	(0.014)
Hispanic	0.001
	(0.010)
Other or 2 +	−0.013
	(0.010)
Age (ref. 18 −29)	
30 −49	−0.040^***^
	(0.008)
50 −69	−0.023^**^
	(0.009)
70 +	0.012
	(0.015)
Enrolled in School (ref. No)	−0.074^***^
	(0.007)
Marital Status (ref. Single)	−0.006
	(0.006)
Has Children (ref. No)	0.032^***^
	(0.007)
Non-English Language at Home (ref. No)	−0.002
	(0.009)
Job Tenure	0.001
	(0.001)
**Baseline Job Quality**	
Hourly Wage	−0.001*
	(0.001)
Schedule Instability Scale	0.006^**^
	(0.002)
Benefits Scale	−0.007^***^
	(0.001)
Observations	34576

Note: Standard errors in parentheses

**Appendix Figure 6:**
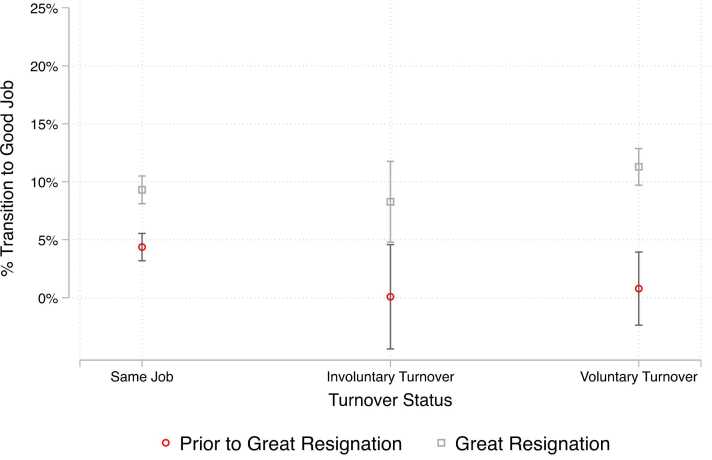
Predicted Probability of Transition to Good Job by Labor Market Tightness and Turnover Status.

## Data Availability

The data underlying this article will be shared on reasonable request to the corresponding author.
